# A comparison of mycophenolate mofetil and calcineurin inhibitor as maintenance immunosuppression for kidney transplant recipients: A meta-analysis of randomized controlled trials

**DOI:** 10.3906/sag-1910-156

**Published:** 2021-06-28

**Authors:** Jin DENG, Yi LU, Lihong HE, Jihong OU, Hongping XIE

**Affiliations:** 1 Department of Health Management, the First Affiliated Hospital of University of South China, Hengyang China; 2 Department of Nephrology, the First Affiliated Hospital of University of South China, Hengyang, China China; 3 Department of Diagnostic Ultrasound, the First Hospital of Changsha China

**Keywords:** Kidney transplantation, mycophenolate mofetil, calcineurin inhibitor, meta-analysis

## Abstract

**Background/aim:**

We conducted a systematic review and meta-analysis of randomized controlled trials (RCTs) to evaluate the comparison and its timing between mycophenolate mofetil (MMF) and calcineurin inhibitor (CNI) as maintenance immunosuppression for kidney transplant recipients.

**Materials and methods:**

The RCTs of MMF versus CNI as maintenance immunosuppression for kidney transplant recipients were searched from PubMed, Embase, Cochrane Central Register of Controlled Trials (CCRCT), and ClinicalTrials.gov. After screening relevant RCTs, two authors independently assessed the quality of included studies and performed a meta-analysis using RevMan5.3. Relative risk (RR) was used to report dichotomous data, while mean difference (MD) with 95% confidence interval (CI) was used to report continuous outcomes. The analysis was conducted using the random-effect model due to the expected heterogeneity among different studies. Four subgroups were allocated to compare MMF with CNI as maintenance immunosuppression: (1) after 3 months of CNI-based therapy, (2) after 6 months of CNI-based therapy, (3) after 12 months of CNI-based therapy, and (4) in recipients with allograft dysfunction.

**Results:**

Twelve RCTs with 950 renal transplant recipients were included. This meta-analysis presented the following results upon comparison between MMF and CNI as maintenance immunosuppression for kidney transplant recipients: (1) MMF significantly improved the glomerular filtration rate (GFR) not only in the comparison performed after 3, 6, or 12 months of CNI-based therapy but also in the comparison of recipients with allograft dysfunction, (2) MMF may increase the risk of acute rejection in the comparison performed after 3 months of CNI-based therapy, but no increase was noted in the comparison performed after 6 or 12 months of CNI-based therapy.

**Conclusion:**

Our present meta-analysis suggested that MMF followed at least 6 months of CNI-based therapy is an effective maintenance immunosuppressive regimen for kidney transplant recipients to improve renal function but not increase rejection.

## 1. Introduction

End-stage renal disease (ESRD) is a chronic, irreversible decline in kidney function that severely and deleteriously affects the duration and quality of life of patients. Approximately 1.9 million patients receive renal replacement therapy (RRT) worldwide [1]. RRT, which includes kidney transplantation (KT), hemodialysis (HD), and peritoneal dialysis (PD), is the only option for individuals with ESRD to survive at present. Compared to dialysis, KT prolongs the life-span, improves renal function and quality of life, and is more cost-effective [2–5]. Nevertheless, a suitable and effective immunosuppressive regimen that minimizes acute rejection (AR) and limits adverse events (AEs) is paramount for KT success. Regarding immunosuppressive therapy, calcineurin inhibitors (CNIs), such as cyclosporine A (CsA) or tacrolimus (TAC), have served as fundamental therapies for renal allograft recipients since CsA became available in the early 1980s. However, significant AEs, such as hypertension, dyslipidemia, new-onset diabetes after transplantation (NODAT), and particularly nephrotoxicity of CNI, have been noted and they serve as major causes of later graft loss [6]. Mycophenolate mofetil (MMF), a prodrug of mycophenolic acid (MPA), which inhibits T and B lymphocyte proliferation, has been shown to reduce the risk of acute allograft rejection and lack nephrotoxicity [7,8]. Moreover, a meta-analysis demonstrated the positive effect of CNI sparing with MMF as solo adjunctive immunosuppressive agents after KT [9]. Several randomized controlled trials (RCTs) compared the outcomes after MMF or CNI withdrawal in renal transplant recipients [10–12]. However, to date, meta-analysis data are not available to compare the efficacy and safety of MMF with CNI as maintenance immunosuppression for kidney transplant recipients. In addition, given the correlation between the duration of CNI and its therapeutic efficacy and side effects, we conducted a systematic review and meta-analysis of RCTs to evaluate the comparison and its timing between MMF and CNI as maintenance immunosuppression for kidney transplant recipients.

## 2. Materials and methods 

### 2.1. Search strategy

PubMed, Embase, Cochrane Central Register of Controlled Trials (CCRCT), and ClinicalTrials.gov were searched without language restrictions using the following mesh terms and entry terms: kidney transplantation, renal transplantations, kidney grafting, mycophenolate mofetil, mycophenolate sodium, cellcept, calcineurin inhibitors, protein phosphatase-2b inhibitors, calcineurin antagonists, cyclosporine, cyclosporine a, tacrolimus, and FK506 (all to September 2019). We retrieved the reference lists of all relevant trials and consulted experts in the field to identify potentially relevant studies.

### 2.2. Inclusion criteria

For inclusion in this meta-analysis, studies had to meet the following criteria: (1) Only RCTs were considered, (2) Patients received renal transplant from a living or deceased donor, (3) Studies compared the outcomes of the use of MMF to CNI as maintenance immunosuppression for kidney transplant recipients, (4) Trials analyzed primary outcomes, including renal function, acute rejection, graft survival, or patient survival. Studies with complete CNI avoidance in de novo patients or multiple organ transplant recipients were excluded. The studies were subsequently allocated to four subgroups to compare MMF and CNI as maintenance immunosuppression: (1) after 3 months of CNI-based therapy, (2) after 6 months of CNI-based therapy, (3) after 12 months of CNI-based therapy; and (4) in recipients with allograft dysfunction.

### 2.3. Study selection 

Two authors separately examined the titles and/or abstracts of each study and excluded irrelevant trials. Subsequently, the full text of all articles was scanned and evaluated independently by two authors strictly according to the inclusion criteria. All disagreements regarding study eligibility for inclusion were discussed to achieve a consensus.

### 2.4. Data extraction

Two authors independently extracted data on the baseline demographic characteristics of participants, study design, intervention and control treatment, and outcome data of studies. We contacted the trial authors or sponsors directly to obtain the required information if data were unavailable. When disagreements occurred, the third author provided an opinion to resolve the issue.

### 2.5. Study quality assessment 

Two authors independently evaluated the quality of the included studies. Disagreements were resolved by consensus. The quality of included studies was evaluated by the Cochrane Handbook [13]. The risk of bias comprised a description and judgment for the following criteria: random sequence generation, allocation concealment, blinding of participants and personnel, blinding of outcome assessment, incomplete outcome data, selective reporting, other source of bias. Each criterion was judged ‘low risk of bias’, ‘unclear risk of bias’, or ‘high risk of bias’. 

### 2.6. Statistical analysis

Outcomes were analyzed using Cochrane Review Manager Software (RevMan5.3, Copenhagen, Denmark: the Nordic Cochrane Centre, the Cochrane Collaboration). Continuous variables are expressed as the mean difference (MD) and 95% confidence interval (CI). The risk ratio (RR) and 95%CI were calculated for dichotomous data. If there are no events in one arm or two arms, the data also will be filled truthfully in the forest figures. The I2-statistic and Chi-squared test were used to assess the heterogeneity of the included studies (I2>50% and p<0.1 indicated significant heterogeneity)[14]. If significant heterogeneity was present among trials, the random-effect model was used. Otherwise, the fixed-effect model was used. Publication bias was evaluated using a funnel plot. 

## 3. Results

### 3.1. Literature selection

**Figure 1:**
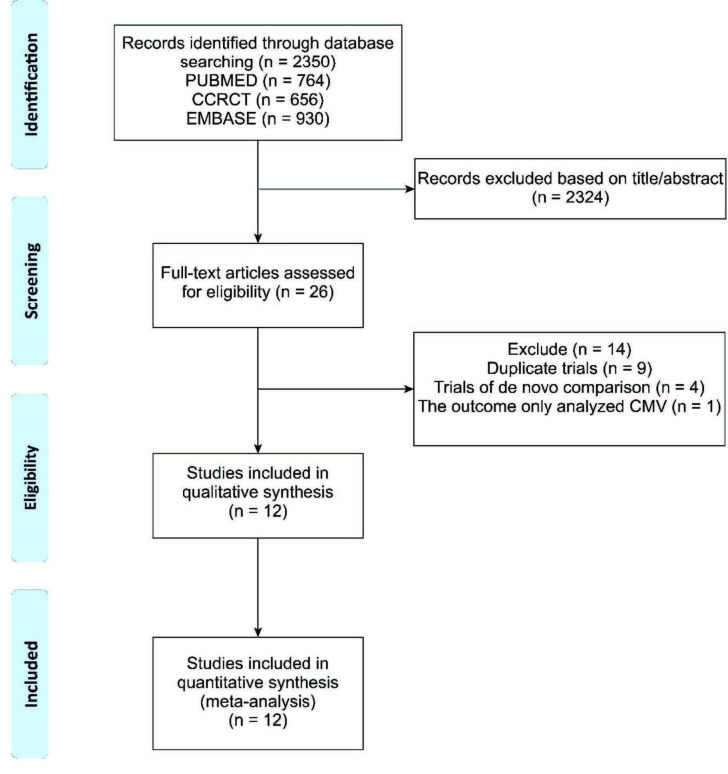
Flow chart of literature selection.

The literature search is presented in Figure 1. A total of 2350 articles were retrieved, and 2324 studies were excluded after examining the titles and abstracts. After reading the full text of the remaining 26 trials, we identified 12 eligible studies for inclusion in the meta-analysis that strictly fulfilled the inclusion and exclusion criteria. Three trials investigated comparison after 3 months of CNI-based therapy [12,15,16], two trials investigated comparison after6 months of CNI-based therapy [11,17], three trials investigated comparison after12 months of CNI-based therapy [10,18,19], and four trials that investigated comparison in recipients with allograft dysfunction [20–23].

### 3.2. Study characteristics and quality assessment

**Figure 2:**
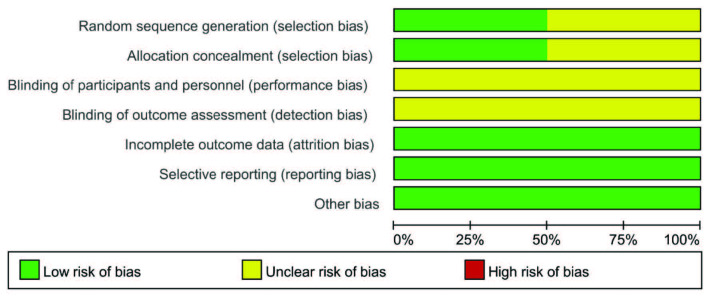
Risk of bias graph: review authors’ judgements about each risk of bias item presented as percentages across all included studies.

A total of 950 eligible renal transplant recipients were included in the meta-analysis, of whom 497 were treated with MMF, and 453 were treated with CNI. All studies reported randomization. Six studies reported random sequence generation and allocation concealment [11,17,18,20,21,23]; however, no studies referred to double-blinding. The baseline characteristics of the included studies are summarized in Table 1, and the risk of bias are showed in Figure 2.

**Table 1:** Baseline characteristics of the included studies.

Subgroup	Study	N	Mean age (years)*		Sex(M/F)	Intervention	Duration(M)
			Recipient	donor			
Comparison after 3 months of CNI-based therapy	Hoerning 2012	T: 6C: 8	T: 46 ± 9.8C: 60 ±11.5	—	T: 2/4C: 3/5	MPA +CsA +Bas+ CS for 3 mo, thenT: EVL+ CS+ MPA (0.72g b.i.d); C: EVL+CS+ Low-CsA (target level:50–75ng/mL)	12
	Hazzan 2005	T: 54C: 54	T: 45.1 ± 11.2C: 42.5 ± 12.1	T: 40.0 ± 14.0C: 36.7 ± 13.1	T: 32/22C: 36/18	MMF+ CsA + ATG+ CS for 3 mo, thenT: CS+ MMF (2g q.d ); C: CS+ CsA (target level:100–300ng/mL)	12
	Schnulle 2002	T: 44C: 40	T: 44.7 ± 13.3C: 51.3 ± 11.5	T: 40.7 ± 15.3C: 47.7 ± 15.4	T: 32/12C: 22/18	MMF+ CsA + CS for 3 mo, thenT: CS+ MMF (1g b.i.d); C: CS+ CsA (target level:100–250ng/mL)	12
Comparison after 6 months of CNI-based therapy	Stevens 2014	T:90C:88	T:47.9 ± 12.1C:46.5 ± 11.6	T: 39.3 ± 13.1C: 42.6 ± 12.1	T: 62/28C: 59/29	TAC+ SRL+ATG+ CS for 6 mo, thenT: SRL+ MMF (1g b.i.d); C: SRL+ TAC (target level:2–4ng/mL)	24
	Mourer 2012	T: 79C: 79	T: 52.5 ± 10.8C: 52.7 ± 13.0	T: 43.3 ± 16.6C: 42.5 ± 14.4	T: 56/23C: 54/25	MMF+ CsA or TAC + CS for 6 mo, thenT: CS+ MMF (AUC:75ug.hr/ml); C: CS+ CsA (AUC3250ng.hr/ml) or TAC (AUC120ng.hr/mL)	36
Comparison after 12 months of CNI-based therapy	Asberg 2013	T: 20C: 19	T: 63.0 ± 11.2C: 56.4 ± 13.4	—	T: 12/8C: 14/5	MMF+ CsA+ CS for 12 mo, thenT: CS+ MMF (2g q.d); C: CS+ CsA (target level:75–125ng/mL)	12
	Albano 2012	T:15C:15	T:58.8 ± 7.6C:62.3 ± 9.5	T: 64.7 ± 12.0C: 62.9 ± 9.8	T: 13/2C: 11/4	CsA +EVL+ CS for 12 mo, thenT: EVL+ CS+ MMF (0.72g b.i.d); C: EVL+ CS+ CsA (target level:200–450ng/mL)	12
	Cransberg 2007	T: 18C: 18	T: 11.9aC: 10.9a	—	T: 8/10C: 14/4	MMF+ CsA+ CS for 12 mo, thenT: CS+ MMF (0.6g b.i.d); C: CS+ CsA (target level:150–200ng/mL)	24
Comparison in allograft dysfunction recipients	Frimat 2006	T:70C: 31	T:43.8 ± 10.6C:44.7 ± 11.1	—	T:55/15C:27/4	T: MMF (2g q.d) +half dose of CsA (target level: not available)C: CsA standard- dose (target level:>80ng/mL)	24
	Dudley 2005	T: 73C: 70	T:43(18–63)bC:43(18–63)b	T:43.8(13-72)bC:34.8(10-65)b	T: 45/28C: 44/26	T:CS+ MMF (2g q.d)C: CsA-based standard therapy (target level:>80ng/mL)	14
	Stoves 2004	T: 13C: 16	—	—	—	T: MMF (1g b.i.d) + reduced dose of CsA (target level:75–100ng/mL)C: CsA standard- dose (target level: unit standard)	6
	Mcgrath 2001	T: 15C: 15	T: 50.4 ± 8.3C: 42.6 ± 3.1	T: 41.8 ± 5.0C: 40.9 ± 2.7	T: 10/5C: 10/5	T: MMF+ CS (2g q.d)C: AZA+ CS+ TAC (target level:8–12ng/mL)	8

### 3.3. Glomerular filtration rate

**Figure 3:**
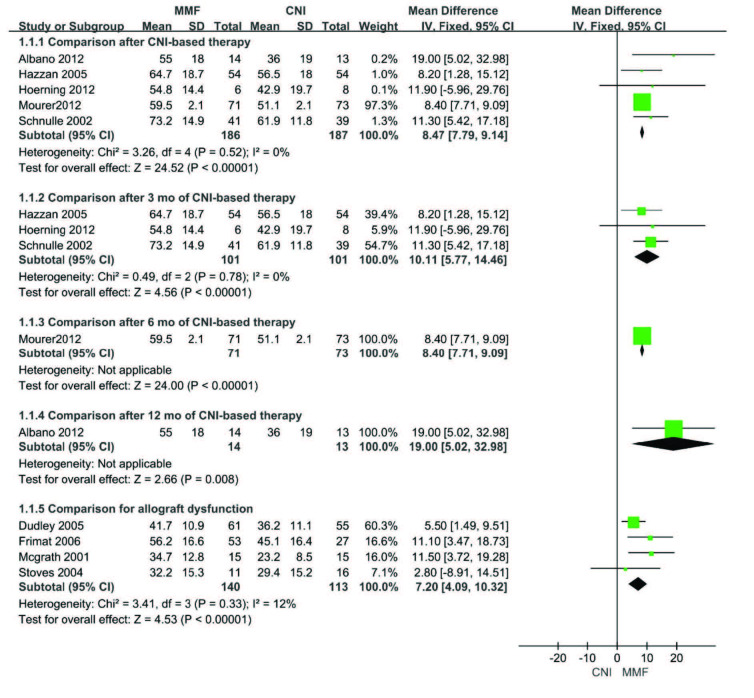
Forest plot of glomerular filtration rate.

Nine studies that reported changes of the GFR were included in the meta-analysis. Compared to CNI, MMF significantly improved the GFR after CNI-based therapy (MD 8.47, 95%CI (7.79, 9.14), p < 0.00001) (Figure 3). Subgroup analysis showed similar effects in comparison after 3, 6, or 12 months of CNI-based therapy (3 months: MD 10.11, 95%CI (5.77, 14.46), p < 0.00001; 6 months: MD 8.40, 95%CI (7.71, 9.09), p < 0.00001 or 12 months: MD 19.00, 95%CI (5.02, 32.98), p = 0.008) (Figure 3). Furthermore, MMF also significantly improved the GFR in comparison of recipients with allograft dysfunction compared with CNI (MD 7.20, 95%CI (4.09, 10.32), p < 0.00001) (Figure 3). 

### 3.4. Graft loss

**Figure 4:**
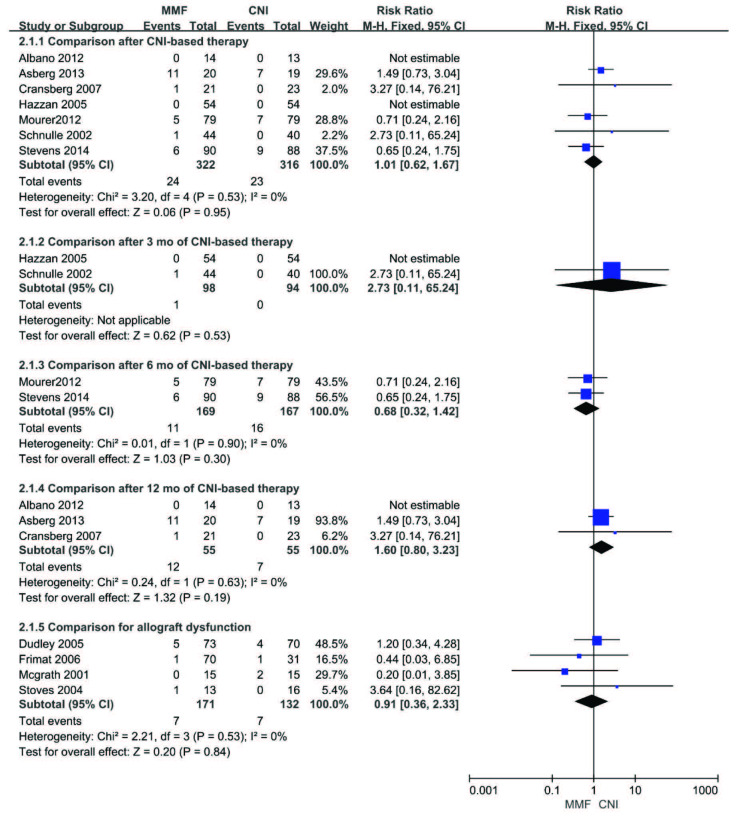
Forest plot of graft loss (including death).

No significant difference in graft loss (including death) was observed between the MMF group and the CNI group after CNI-based therapy (RR 1.01, 95%CI (0.62, 1.67), p = 0.95). Subgroup analysis showed similar effects in comparison after 3, 6, or 12 months of CNI-based therapy (3 months: RR 2.73, 95%CI (0.11, 65.24), p = 0.53; 6 months: RR 0.68, 95%CI (0.32, 1.42), p = 0.30 or 12 months: RR 1.60, 95%CI (0.80, 3.23), p = 0.19). Similar effect was also seen in comparison of recipients with allograft dysfunction (RR 0.91, 95%CI (0.36, 2.33), p = 0.84). The fixed-effect model was used for the meta-analysis given that no heterogeneity was noted among the included studies. One study was excluded for analysis due to the absence of graft loss data [15]. The results are presented in Figure 4.

### 3.5. Mortality 

**Figure 5:**
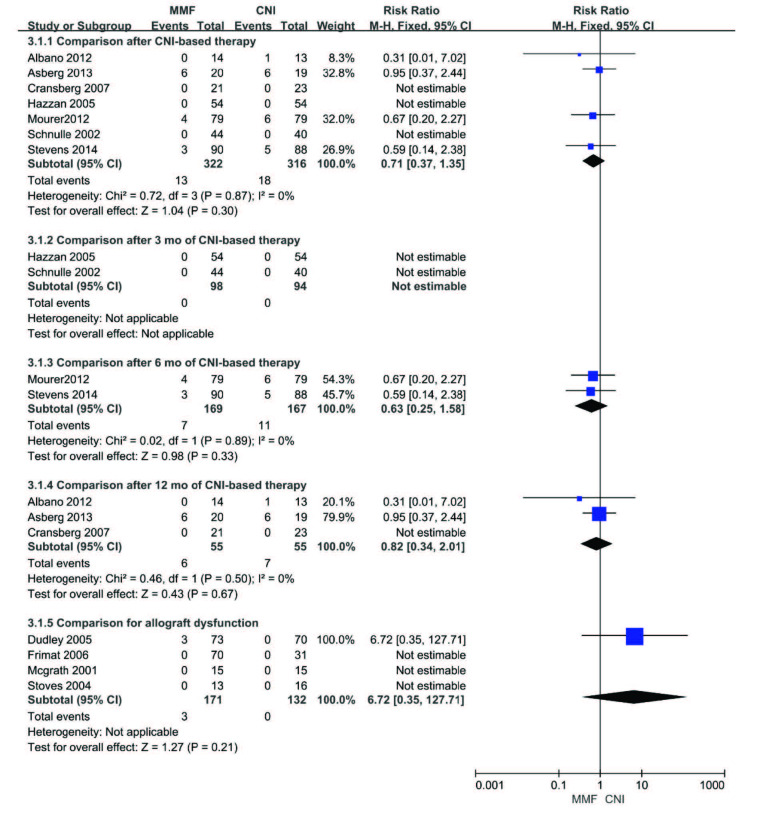
Forest plot of mortality.

Eleven included studies reported mortality data. There were no significant differences in mortality between the MMF and CNI groups after CNI-based therapy (RR 0.71, 95%CI (0.37, 1.35), p = 0.30). Subgroup analysis showed similar effects in comparison after 3, 6, or 12 months of CNI-based therapy (3 months: could not be estimated; 6 months: RR 0.63, 95%CI (0.25, 1.58), p = 0.33 or 12 months: RR 0.82, 95%CI (0.34, 2.01), p = 0.67). Moreover, there was also no significant difference in mortality between the MMF and CNI groups in comparison of recipients with allograft dysfunction (RR 6.72, 95%CI (0.35, 127.71), p = 0.21). The fixed-effect model was used given the lack of heterogeneity among the studies. The results are presented in Figure 5.

### 3.6. Acute rejection 

**Figure 6:**
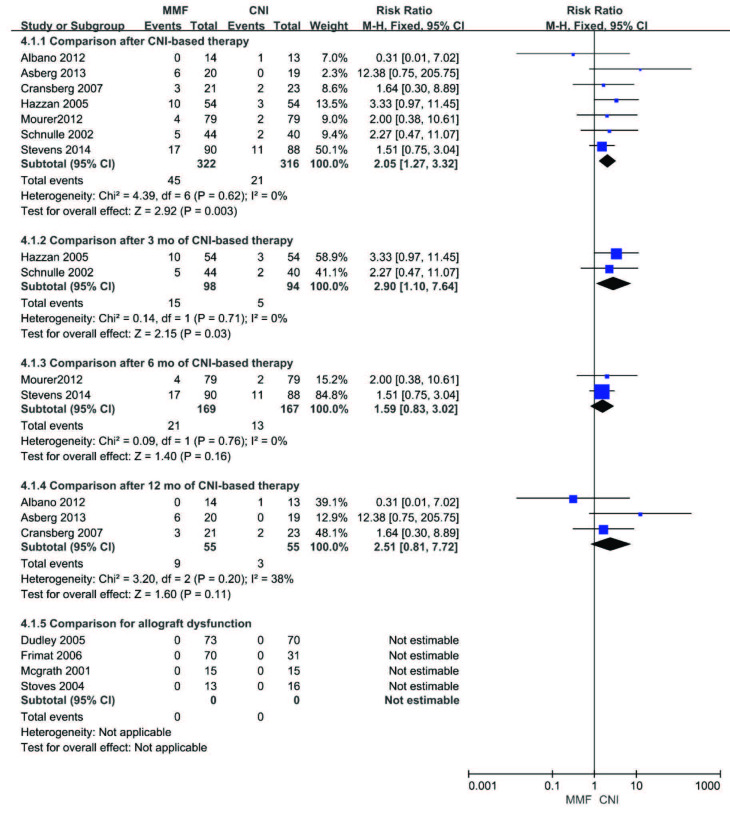
Forest plot of acute rejection (biopsy proven).

MMF was associated with increased episodes of acute rejection (biopsy proven) compared with CNI after CNI-based therapy (RR 2.05, 95%CI (1.27, 3.32), p = 0.003). Similar effect was seen in comparison after 3 months of CNI-based therapy (RR 2.90, 95%CI (1.10, 7.64), p = 0.03) when subgroup analysis was performed. However, no significant differences in acute rejection were found between the MMF and CNI groups for comparison after 6 or 12 months of CNI-based therapy (6 months: RR 1.59, 95%CI (0.83, 3.02), p = 0.16 or 12 months: RR 2.51, 95%CI (0.81, 7.72), p = 0.11). No acute rejection episodes occurred in recipients with allograft dysfunction. The fixed-effect model was used given the lack of heterogeneity among the studies. The results are presented in Figure 6.

### 3.7. Adverse events

A comparison of adverse events in the MMF and CNI groups is shown in Table 2. The random-effect model was used if significant heterogeneity (I2>50% and p < 0.1) was presented among studies. Otherwise, the fixed-effect model was used instead. The results indicated that MMF reduced the occurrence rate of proteinuria (RR 0.63, 95%CI (0.43, 0.92), p = 0.02), although the opposite effects were presented for anemia (RR 2.36, 95%CI (1.46, 3.81), p = 0.0005) and diarrhea (RR 5.36, 95%CI (2.66, 10.80), p = 0.00001). The incidence rates of infection, NODAT, malignancies, and hypertension were similar between the MMF and CNI groups. 

**Table 2:** Summary of adverse events of included studies comparing MMF with CNI groups as maintenance immunosuppression after kidney transplantation.

Outcome	Studies	MMF group	CNI group	Heterogeneity(P, I2)	Statistical method	Effect estimate	P value
Infection	7	156/384	117/339	0.006, 66%	Risk ratio(M-H, Random, 95%CI)	1.19(0.83, 1.73)	0.34
Anemia	5	56/250	20/211	0.61, 0%	Risk ratio(M-H, Fixed, 95%CI)	2.36 (1.46, 3.81)	0.0005
Diarrhea	5	54/281	8/235	0.32, 15%	Risk ratio(M-H, Fixed, 95%CI)	5.36 (2.66, 10.80)	0.00001
NODAT	5	25/241	28/238	0.77, 0%	Risk ratio(M-H, Fixed, 95%CI)	0.86 (0.53, 1.42)	0.56
Malignancies	4	12/254	13/198	0.77, 0%	Risk ratio(M-H, Fixed, 95%CI)	0.84 (0.39, 1.84)	0.66
Proteinuria	3	34/139	30/100	0.38, 0%	Risk ratio(M-H, Fixed, 95%CI)	0.63 (0.43, 0.92)	0.02
Hypertension	2	5/88	11/85	0.35, 0%	Risk Ratio(M-H, Fixed, 95%CI)	0.46 (0.17, 1.23)	0.12

### 3.8. Publication bias

**Figure 7:**
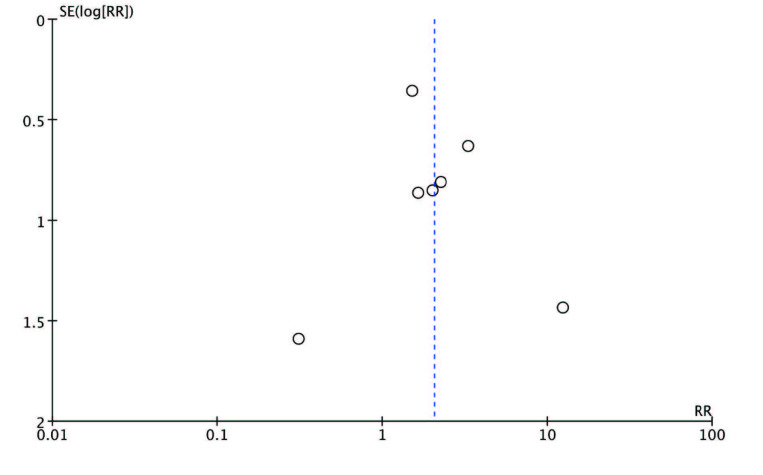
Funnel plot for acute rejection.

A funnel plot of acute rejection was examined to evaluate publication bias. As shown in Figure 7, no publication bias was observed. 

## 4. Discussion

Kidney transplantation, which is a form of RRT, is an efficient and preferable option for ESRD patients [3]. However, acute rejection and graft loss represent the clinical concerns after kidney transplantation (KT). Thus, safe and effective immunosuppressive therapy is needed to reduce graft failure caused by acute rejection and CNI-related nephrotoxicity in the most prevalent CNI-based immunosuppressive regimes [24, 25]. As a nonnephrotoxic immunosuppressive drug, MMF improves renal function without acute rejection after CNI withdrawal [26–28]. Moreover, two studies reported that MMF could have nephroprotective properties [29,30]. Recently, a meta-analysis suggested that CNI sparing strategies with adjunctive MMF after KT can improve renal function, possibly reduce graft loss, and increase rejection rates only after elective CNI elimination [9]. Thus, MMF may enhance renal function but not increase rejection and nephrotoxicity, consequently improving patient and graft survival.

This is the first meta-analysis to evaluate the comparison and its timing between MMF and CNI as maintenance immunosuppression for kidney transplant recipients. We analyzed the data of 12 studies that compared the use of MMF and CNI as maintenance immunosuppression for kidney transplant recipients. The results of our present meta-analysis indicate that MMF significantly improved the GFR not only in the comparison performed after 3, 6, or 12 months of CNI-based therapy but also in the comparison of recipients with allograft dysfunction. This result suggested the ongoing benefits of using MMF instead of CNI not only in patients with deteriorating renal function but also in patients with stable renal function after KT regardless of the timing of the alternative. Interestingly, our present meta-analysis also found that MMF may increase the risk of acute rejection in the comparison performed after 3 months of CNI-based therapy, but no increase was noted in the comparison performed after 6 or 12 months of CNI-based therapy. Taken together, the results of this analysis indicate that MMF offers similar efficiency as CNI after at least 6 months of CNI-based therapy as maintenance immunosuppression for kidney transplant recipients, while MMF appears safer than CNI, as reflected by its protective effects on renal function. However, this finding must be further demonstrated by more large-scale, high-quality, and long-term studies. In addition, MMF is associated with a reduced incidence of proteinuria, whereas the opposite effects were noted for anemia and diarrhea compared to CNI.

Several limitations to this meta-analysis should be noted. Above all, most of the included trials had small samples and were not multicenter RCTs. In addition, no studies were double-blinded. Furthermore, data from some studies were unavailable or deficient and could not be obtained from the original authors, which may weaken the evidence of the results. Moreover, given that a few studies in each subgroup and several studies with a short duration, the efficacy and safety of MMF for renal transplant recipients must be proven by further large-scale and long-term studies. Finally, some heterogeneity in clinical features, such as the immunosuppressive therapy and drug dosages, was noted; however, the included studies had similar baseline characteristics. Thus, more large-scale, high-quality, and multicenter RCTs with longer duration times and reduced heterogeneity are required to address the above limitations.

In conclusion, the result of our present meta-analysis is that MMF offers similar efficiency as CNI after at least 6 months of CNI-based therapy as maintenance immunosuppression for kidney transplant recipients, while MMF appears safer than CNI, as reflected by its protective effects on renal function. It is suggested that MMF followed at least 6 months of CNI-based therapy is an effective maintenance immunosuppressive regimen for kidney transplant recipients to improve renal function but not increase rejection. However, these results must be confirmed in future studies. 

## Informed consent

Not required.
